# Dynamical property of interaction solutions to the Chafee-Infante equation via NMSE method

**DOI:** 10.1016/j.heliyon.2024.e36168

**Published:** 2024-08-13

**Authors:** Mohammad Mobarak Hossain, Sushika Akter, Md. Mamunur Roshid, Harun-Or- Roshid, Md. Abu Naim Sheikh

**Affiliations:** a Department of Mathematics, Sunamgonj Science and Technology University, Bangladesh; b Department of Mathematics, Dhaka University of Engineering & Technology, Gazipur, Bangladesh; c Department of Mathematics, Hamdard University Bangladesh, Bangladesh; d Department of Mathematics, Pabna University of Science and Technology, Bangladesh

**Keywords:** Chafee-infante equation, New modified simple equation method, Conformable fractional derivative, Multi-soliton solution, Interaction solution

## Abstract

In this work, we study the Chafee-Infante model with conformable fractional derivative. This model describes the energy balance between equator and pole of solar system, which transmit energy via heat diffusion. To explore the multi soliton solutions and their interaction, we implemented the new modified simple equation (NMSE) scheme. Under some conditions, the obtained solutions are trigonometric, hyperbolic, exponential and their combine form. Only the proposed technique can be provided the solution in terms of trigonometric and hyperbolic form together directly. The periodic, solitary wave and novel interaction of such solitary and sinusoidal solutions has also been established and discussed analytically. For the special values of the existing free parameter, some novel waveforms are existed for the proposed model including, periodic solution, double periodic wave solution, multi-kink solution. The behavior of the obtained solutions is presented in 3-D plot, density plot and counter plot with the help of computational software Maple 18.

## 1 Introduction

Majority of modeling for natural serious nonlinear behaviors in this world can be illustrated by the nonlinear partial differential equations (NPDEs). To visualize the internal mechanism and properties of the models even natural happenings related to the models in both visible and non-visible effects, derivations of analytical solutions of the corresponding model are essential facts. The solutions to NPDEs are very much important due to their wide range of applications in physical sciences and engineering branches such as fluid mechanic, plasma physics, optical fibers, signal processing, mechanical engineering etc. A number of efficient and powerful techniques have been ascertained to extract analytical solution as well as to illustrate the dynamical behavior of the NPDEs. The $\left(G^{\prime }/G\right)$ -expansion method [1], Bright and dark optical solitons method [2], JEF method [3], SE method [4], the AD scheme [5], the MSE method [6], extension exponential rational function method [7], Hirota bilinear method [8], EMSE method [9] the Kudryashov's scheme [10,11], sine-Gordon expansion scheme [12], Lie–Bäcklund symmetries [13], Wronskian approach [14], the generalized exponential rational function [15], canonical-like transformation [16], exp-function method [17,18], Extended (G'/G) -Expansion method [19], improved (G′/G)-expansion approach [20], exp (−φ(ξ))-expansion method [21], Inverse-engineering structure [22], extended tanh method [23,24], EDAM method [25], IME tanh-function technique [[26], [27], [28]], ESE method [29], NMSE method [30], Hirota direct method [31], MEM method [32], and similar more are developed to extract solutions of NPDEs. Most of the above techniques can provide only single traveling wave solutions of nonlinear models, but the Wronskian, Hirota bilinear [33,34] and NMSE can derive multi-soliton and multi-wave solutions. The two techniques (Wronskian, Hirota bilinear) are very old and not suitable for the model which has no bilinear formation. Alongside this, NMSE technique is a recent innovative technique and can be applied on the model which has no bilinear formation. Owing to this fact, we choose the procedure to integrate nonlinear model to extract mutli-wave and multi-soliton solutions.

Based on the foregoing, the newly proposed solution of the Chafee-Infante equation (CI) equation is recovered using the NMSE method with conformable fractional derivative. As far as we are aware, this is the first time we have studied the analytical solution of the CI problem using this method. With the use of the new soliton solution, graphical representations, and thorough analysis, we examine the CI equation's dynamic behavior. The analytical answers demonstrate that the new approach can reconstruct the precise solution of the CI problem with a smaller sample size, faster convergence, and superior simulation results.

We will develop an algorithm for creating a multiple-wave soliton solution for in this chapter. Here we start the (1 + 1)-dimensional CI Model [35,36] as:

(1) $$D_{t}^{\zeta }u-u_{xx}-\alpha u+\alpha u^{3}=0\text{.}$$

where 0<ζ≤1. The processes to find the solutions of this equation are the same as that has shown in the reference paper [13,15] and so on.

The main motivation of this study is investigated the multi soliton solutions and their interaction of fractional (1 + 1)-dimensional CI model by using new formation of modified simple equation method. The limitation of this technique is that, it cannot solve all nonlinear models through the system is integrable. The calculation is also complicated if the balance number is more than two.

The arrangement of the work is followed: In division two, some feature of fractional derivative and explore the work rule of NMSE technique; In division three, trail the NMSE technique to fractional CI model; In division four, discus the numerical form of the obtained solutions and show the fractional effect with three and density diagram; In section five, we compare this work with others existing work; In division six, the overview of this work.

## 2 Preliminaries and methodology

Fractional derivatives extend traditional calculus, offering enhanced modeling of nonlinear evolution equations (NLEEs). Unlike integer-order derivatives, they capture memory and hereditary effects, crucial for systems where the future state depends on past behaviors. This non-local property makes them ideal for describing complex dynamics in physical, biological, and financial systems [[37], [38], [39], [40]]. In anomalous diffusion, viscoelastic materials, and fractal-like phenomena, fractional derivatives outperform classical models by accommodating irregular patterns and scaling laws. They allow for greater flexibility in initial and boundary conditions, enhancing the fit to empirical data and improving predictive accuracy. This versatility spurs advancements in numerical techniques for solving differential equations, leading to more precise and stable solutions. Conformable derivative is one of the fractional derivatives, that's offering a more straightforward generalization, preserve essential properties like the chain rule, making them easier to apply while still capturing essential system behaviors. Moreover, conformable derivatives provide a robust framework for accurately modeling and analyzing NLEEs across various scientific and engineering fields [10,[41], [42], [43], [44]], capturing intricate system behaviors that traditional methods cannot.

### 2.1 Conformable derivative

Initially Khalil [45] and T. Abdeljawad [46] introduce such derivative as:

For F:(0,∞)→R, the fractional ζ order conformable derivatives defined by:

$$D_{t}^{\zeta }f\left(t\right)=\lim_{h\to 0}\left[\frac{F\left(t+\varepsilon t^{1-\zeta }\right)-F\left(t\right)}{h}\right]for\;all\;0<\zeta \leq 1,0<t\text{.}$$

From the following theorem, conformable fractional derivatives [[41], [42], [43], [44]] are easily understood.

Theorem 1 For ζ∈(0,1], G=G(t) and F=F(t),ζ− conformable differentiable [47] with t>0:

(i) $D_{t}^{\zeta }\left(\text{cF}+\text{dG}\right)=cD_{t}^{\zeta }F+dD_{t}^{\zeta }G$, for all d,c∈R.
(ii) $D_{t}^{\zeta }\left(t^{\alpha }\right)=\alpha t^{\alpha -\zeta }$, ∀α∈R.
(iii) $D_{t}^{\zeta }\left(\text{FG}\right)=FD_{t}^{\zeta }\left(G\right)+GD_{t}^{\zeta }\left(F\right)\text{.}$ :
(iv) $D_{t}^{\zeta }\left(F/G\right)=\frac{GD_{t}^{\zeta }\left(F\right)-FD_{t}^{\zeta }\left(G\right)}{G^{2}}\text{.}$ :

For differentiable F, $D_{t}^{\zeta }\;\left(F\left(t\right)\right)=t^{1-\zeta }\frac{\text{dF}}{\text{dt}}$.

Theorem 2 *If*S:(0,∝)→R*be real valued function as*S*be*ζ*-conformable differentiable* [48] *and* H *be a differentiable with same range*, *then*:

$$D_{t}^{\zeta }\left(SoH\right)\left(t\right)=t^{1-\zeta }{H\left(t\right)}^{\zeta -1}H^{\prime }\left(t\right){D_{t}^{\zeta }\left(S\left(t\right)\right)}_{t=H\left(t\right)}$$

Where $H^{\prime }=\frac{dH}{dt}$:

### 2.2 NMSE method

In this subdivision, we explore the new modified simple equation (NMSE) technique to solve NLEEs. The main advantages are investigated the multi soliton solution and diverse type of interaction soliton solution. Most of the above techniques can provide only single traveling wave solutions of nonlinear models, but the Wronskian, Hirota bilinear [33,34] and NMSE can derive multi-soliton and multi-wave solutions. The two techniques (Wronskian, Hirota bilinear) are very old and not suitable for the model which has no bilinear formation.

For NMSE Technique [30], consider:

(2) $$G\left(h,h_{xx},h_{t},h_{xt}\;\ldots \ldots \ldots \right)=0\text{.}$$

where h=h(x,t), G be polynomial of h and derivatives of h. The following stages can be used to explain the NMSE approach in its entirety.

Step-01 : Trial solution of eq. (2) will be:

(3) $$h=\sum_{r=0}^{M}\sum_{i+j=r}^{M}\left[\alpha_{j}{\left(\frac{f_{2}^{\prime }\left(\eta_{2}\right)}{f_{2}\left(\eta_{2}\right)}\right)}^{j}\times \alpha_{i}{\left(\frac{f_{1}^{\prime }\left(\eta_{1}\right)}{f_{1}\left(\eta_{1}\right)}\right)}^{i}\right].\;$$

Where $\eta_{i}=k_{i}x-\omega_{i}t,i=1,2$ and $\alpha_{i}{,\alpha }_{j}$ with i,j=0,1,2…. are constant. $\alpha_{M}\neq 0\text{,}$ the unknown functions $f_{1}\left(\eta_{1}\right)$ and $f_{2}\left(\eta_{2}\right)$ to be determine later.

Step-02 : To control M, we balance the highest-order derivatives and the nonlinear factor in eq. (2).

Step-03 : Substituting h(x,t) in eq. (2), we obtain a polynomial of $f_{1}^{i},{\;f}_{2}^{j}$ and $f_{1}^{-i}f_{2}^{-j}$.

Step-04 : Comparing the co-efficient with same power of $f_{1}^{i}f_{2}^{j}$ and solving these, $\alpha_{i}{,\alpha }_{j},f_{1}$ and $f_{2}$ can obtained.

RemarkIn the modified simple equation method [4,6], the solution of eq. (2) is considered the following form:

$$u=\sum_{i=0}^{M}\alpha_{i}{\left(\frac{f_{1}^{\prime }\left(\eta_{1}\right)}{f_{1}\left(\eta_{1}\right)}\right)}^{i}$$

Where $\eta_{1}=\text{kx}-\omega t$ and $\alpha_{i}$ with i=0,1,2…. are arbitrary constant to be resolute later such that $\alpha_{M}\neq 0$ the functions $f_{1}\left(\eta_{1}\right)$ is an unknown function.

## 3 Multi-soliton solution of CI model by NMSE technique

In this section, we inject the NMSE scheme on fractional CI [9] model as:

(4) $$D_{t}^{\zeta }u-u_{\text{xx}}-\alpha u+\alpha u^{3}=0\text{.}$$

According to NMSE scheme, the solution of equation (4) becomes,

(5) $$u\left(x,t\right)=a_{0}+a_{1}\left[\frac{f_{1}^{\prime }\left(\eta_{1}\right)}{f_{1}\left(\eta_{1}\right)}\right]+a_{2}\left[\frac{f_{2}^{\prime }\left(\eta_{2}\right)}{f_{2}\left(\eta_{2}\right)}\right]\text{.}$$

where $\eta_{1}=k_{1}x-\omega_{1}\frac{t^{\zeta }}{\zeta }$, $\eta_{2}=k_{2}x-\omega_{2}\frac{t^{\zeta }}{\zeta }\text{;}$ $k_{1},k_{2}$ are angular wave numbers and $\omega_{1},\omega_{2}$ are the waves frequency, $a_{0},a_{1},a_{2}$ are arbitrary constants such that $a_{2},a_{1}\neq 0\text{,}$ $f_{1}\left(\eta_{1}\right)$ and $f_{2}\left(\eta_{2}\right)$ are unknown function to be determine later. For simplification, we uniformly consider as:

$$f_{2}\left(\eta_{2}\right)\equiv f_{2},{\;f}_{1}\left(\eta_{1}\right)\equiv f_{1}\;\text{and}\;f^{\prime }\equiv \frac{df}{d\eta }$$

Now we substitute the necessary derivative of u(x,t) in eq. (4). Then we get a polynomial.

(6) $${-a}_{1}\omega_{1}f_{1}^{\prime \prime }-a_{1}k_{1}^{2}f_{1}^{\prime \prime \prime }-\;\alpha a_{1}f_{1}^{\prime }+3\alpha a_{0}^{2}a_{1}f_{1}^{\prime }=0\text{.}$$

(7) $${-a}_{1}\omega_{1}f_{1}^{\prime \prime }-a_{1}k_{1}^{2}f_{1}^{\prime \prime \prime }-\;\alpha a_{1}f_{1}^{\prime }+3\alpha a_{0}^{2}a_{1}f_{1}^{\prime }=0\text{.}$$

(8) $${-a}_{2}\omega_{2}f_{2}^{\prime }-a_{2}k_{2}^{2}f_{2}^{\prime \prime \prime }-\alpha a_{2}f_{2}^{\prime }+3\alpha a_{2}a_{0}^{2}f_{2}^{\prime }=0\text{.}$$

(9) $$a_{1}\omega_{1}f_{1}^{\prime 2}+{3a}_{1}k_{1}^{2}f_{1}^{\prime }f_{1}^{\prime \prime }+3\alpha a_{0}a_{1}^{2}f_{1}^{\prime 2}=0\text{.}$$

(10) $$a_{2}\omega_{2}f_{2}^{\prime 2}+3a_{2}k_{2}^{2}f_{2}^{\prime }f_{2}^{\prime \prime }+3\alpha a_{0}a_{2}^{2}f_{2}^{\prime 2}=0\text{.}$$

(11) $$-2a_{1}k_{1}^{2}f_{1}^{\prime 3}+\alpha a_{1}^{3}f_{1}^{\prime 3}=0\text{.}$$

(12) $$-2a_{2}k_{2}^{2}f_{2}^{\prime 3}+\alpha a_{2}^{3}f_{2}^{\prime 3}=0\text{.}$$

(13) $$6\alpha a_{0}a_{1}a_{2}f_{1}^{\prime }f_{2}^{\prime }=0\text{.}$$

(14) $$3\alpha a_{1}a_{2}^{2}f_{1}^{\prime }f_{2}^{\prime 2}=0\text{.}$$

(15) $$3\alpha a_{1}a_{2}^{2}f_{1}^{\prime }f_{2}^{\prime 2}=0$$

From eq. (10) to eq. (15) we find that

$$a_{0}=0,1,-1;\;a_{1}=0,\pm \sqrt{\frac{2}{\alpha }}k_{1};\;a_{2}=0,\pm \sqrt{\frac{2}{\alpha }}k_{2}$$

**Case-I:** when $a_{0}=0$ then eq. (7) to eq. (9) gives us:

(16) $$f_{1}^{\prime }=-\frac{3k_{1}^{2}f_{1}^{\prime \prime }}{\omega_{1}}\text{.}$$

(17) $$f_{2}^{\prime }=-\frac{3k_{2}^{2}f_{2}^{\prime \prime }}{\omega_{2}}\text{.}$$

Putting eq. (16) in eq. (6) and eq. (17) in eq. (7) then we get,

(18) $$f_{1}=\frac{C_{1}3k_{1}^{4}}{{\omega_{1}}^{2}-3k_{1}^{2}\alpha }\;e^{\theta \eta_{1}}+C_{2}\text{.}$$

(19) $$f_{2}=\frac{C_{3}3k_{2}^{4}}{{\omega_{2}}^{2}-3k_{2}^{2}\alpha }\;e^{\emptyset \eta_{2}}+C_{4}\text{.}$$

where $\theta =\frac{3k_{1}^{2}\alpha -{\omega_{1}}^{2}}{\omega_{1}k_{1}^{2}}$ and $\emptyset =\frac{3k_{2}^{2}\alpha -\omega_{2}^{2}}{\omega_{2}k_{2}^{2}}$:

Now from eq. (5) with the help of eq. (18) and eq. (19) for $a_{1}=\pm \sqrt{\frac{2}{\alpha }}k_{1}$, $a_{2}=\pm \sqrt{\frac{2}{\alpha }}k_{2}$ we get,

(20) $$u\left(x,t\right)=\pm \frac{\sqrt{\frac{2}{\alpha }}{{3k}_{1}}^{3}C_{1}e^{\theta \eta_{1}}}{\frac{C_{1}3k_{1}^{4}}{{\omega_{1}}^{2}-3k_{1}^{2}\alpha }\;e^{\theta \eta_{1}}+C_{2}}\mp \frac{\sqrt{\frac{2}{\alpha }}{{3k}_{2}}^{3}C_{3}e^{\emptyset \eta_{2}}}{\frac{C_{3}3k_{2}^{4}}{{\omega_{2}}^{2}-3k_{2}^{2}\alpha }\;e^{\emptyset \eta_{2}}+C_{4}}\text{.}$$

where $\theta =\frac{3k_{1}^{2}\alpha -{\omega_{1}}^{2}}{\omega_{1}k_{1}^{2}},\emptyset =\frac{3k_{2}^{2}\alpha -\omega_{2}^{2}}{\omega_{2}k_{2}^{2}}$ and $\omega_{1},k_{1},C_{1},C_{2},\omega_{2},k_{2},C_{3},C_{4}$ are arbitrary constants.

With $C_{1}=\pm \frac{\frac{1}{3}{\omega_{1}}^{2}-\alpha k_{1}^{2}}{k_{1}^{4}},C_{3}=\pm \frac{{{\frac{1}{3}\omega }_{2}}^{2}-k_{2}^{2}\alpha }{k_{2}^{4}},C_{2}=C_{4}=1$ we get from eq. (20),

(21) $$u\left(x,t\right)=\frac{{\omega_{1}}^{2}-3k_{1}^{2}}{\sqrt{2\alpha }k_{1}\omega_{1}}\mathrm{sech}\left(\frac{\theta \eta_{1}}{2}\right)e^{\frac{\theta \eta_{1}}{2}}+\frac{{\omega_{2}}^{2}-3k_{2}^{2}}{\sqrt{2\alpha }k_{2}\omega_{2}}\mathrm{sech}\left(\frac{\emptyset \eta_{2}}{2}\right)e^{\frac{\emptyset \eta_{2}}{2}}\text{.}$$

If $C_{1}=\pm \frac{\frac{1}{3}{\omega_{1}}^{2}-\alpha k_{1}^{2}}{k_{1}^{4}},C_{3}=\pm \frac{{{\frac{1}{3}\omega }_{2}}^{2}-k_{2}^{2}\alpha }{k_{2}^{4}},C_{2}=-1$ and $C_{4}=1$ we get from eq. (20) as,

(22) $$u\left(x,t\right)=-\frac{{\omega_{1}}^{2}-3k_{1}^{2}}{\sqrt{2\alpha }k_{1}\omega_{1}}\text{cosech}\left(\frac{\theta \eta_{1}}{2}\right)e^{-\frac{\theta \eta_{1}}{2}}+\frac{{\omega_{2}}^{2}-3k_{2}^{2}}{\sqrt{2\alpha }k_{2}\omega_{2}}\mathrm{sech}\left(\frac{\emptyset \eta_{2}}{2}\right)e^{\frac{\emptyset \eta_{2}}{2}}\text{.}$$

If $C_{1}=\pm \frac{\frac{1}{3}{\omega_{1}}^{2}-\alpha k_{1}^{2}}{k_{1}^{4}},C_{3}=\pm \frac{{{\frac{1}{3}\omega }_{2}}^{2}-k_{2}^{2}\alpha }{k_{2}^{4}},C_{2}=C_{4}=-1$ we get from eq. (20),

(23) $$u\left(x,t\right)=-\frac{{\omega_{1}}^{2}-3k_{1}^{2}}{\sqrt{2\alpha }k_{1}\omega_{1}}\text{cosech}\left(\frac{\theta \eta_{1}}{2}\right)e^{-\frac{\theta \eta_{1}}{2}}-\frac{{\omega_{2}}^{2}-3k_{2}^{2}}{\sqrt{2\alpha }k_{2}\omega_{2}}\text{cosech}\left(\frac{\emptyset \eta_{2}}{2}\right)e^{-\frac{\emptyset \eta_{2}}{2}}\text{.}$$

Where $\theta =\frac{3k_{1}^{2}\alpha -{\omega_{1}}^{2}}{\omega_{1}k_{1}^{2}},\emptyset =\frac{3k_{2}^{2}\alpha -\omega_{2}^{2}}{\omega_{2}k_{2}^{2}}$ and $\omega_{1},k_{1},\omega_{2},k_{2}$ are arbitrary constants.

**Case-II:** For $a_{0}=1$ then the remaining equations is gives us:

(24) $$f_{1}^{\prime }=-\frac{3k_{1}^{2}f_{1}^{\prime \prime }}{\omega_{1}+3\alpha a_{1}}\text{.}$$

(25) $$f_{2}^{\prime }=-\frac{3k_{2}^{2}f_{2}^{\prime \prime }}{\omega_{2}+3\alpha a_{2}}\text{.}$$

Putting eq. (24) in eq. (6) and eq. (25) in eq. (7) then we get,

(26) $$f_{1}=\frac{C_{5}3k_{1}^{4}}{{\omega_{1}}^{2}+3\alpha a_{1}\omega_{1}+6\alpha k_{1}^{2}}\;e^{m\eta_{1}}+C_{6}\text{.}$$

(27) $$f_{2}=\frac{C_{7}3k_{2}^{4}}{{\omega_{2}}^{2}+3\alpha a_{2}\omega_{2}+6\alpha k_{2}^{2}}\;e^{n\eta_{2}}+C_{8}\text{.}$$

where $m=-\frac{{\omega_{1}}^{2}+3\alpha a_{1}\omega_{1}+6\alpha k_{1}^{2}}{k_{1}^{2}\left(\omega_{1}+3\alpha a_{1}\right)}$ and $n=-\frac{{\omega_{2}}^{2}+3\alpha a_{2}\omega_{2}+6\alpha k_{2}^{2}}{k_{2}^{2}\left(\omega_{2}+3\alpha a_{2}\right)}$.

Now from eq. (5) with the help of eq. (26) and eq. (27) for $a_{1}=\pm \sqrt{\frac{2}{\alpha }}k_{1}$, $a_{2}=\pm \sqrt{\frac{2}{\alpha }}k_{2}$ we get,

(28) $$u\left(x,t\right)=1\mp \frac{\sqrt{\frac{2}{\alpha }}\frac{C_{5}3k_{1}^{3}}{\omega_{1}+3\alpha a_{1}}e^{m\eta_{1}}}{\frac{C_{5}3k_{1}^{4}}{{\omega_{1}}^{2}+3\alpha a_{1}\omega_{1}+6\alpha k_{1}^{2}}\;e^{m\eta_{1}}+C_{6}}\mp \frac{\sqrt{\frac{2}{\alpha }}\frac{C_{7}3k_{2}^{3}}{\omega_{2}+3\alpha a_{2}}e^{n\eta_{2}}}{\frac{C_{7}3k_{2}^{4}}{{\omega_{2}}^{2}+3\alpha a_{2}\omega_{2}+6\alpha k_{2}^{2}}\;e^{n\eta_{2}}+C_{8}}\text{.}$$

where $m=-\frac{{\omega_{1}}^{2}+3\alpha a_{1}\omega_{1}+6\alpha k_{1}^{2}}{k_{1}^{2}\left(\omega_{1}+3\alpha a_{1}\right)}$, $n=-\frac{{\omega_{2}}^{2}+3\alpha a_{2}\omega_{2}+6\alpha k_{2}^{2}}{k_{2}^{2}\left(\omega_{2}+3\alpha a_{2}\right)}$ and $\omega_{1},k_{1},C_{5},C_{6},\omega_{2},k_{2},C_{7}\text{,}$ :

$C_{8}$ are arbitrary constants.

Under the condition $C_{5}=\frac{\frac{1}{3}{\omega_{1}}^{2}+a_{1}\omega_{1}\alpha +2k_{1}^{2}\alpha }{k_{1}^{4}},C_{6}=1,C_{7}=\frac{\frac{1}{3}{\omega_{2}}^{2}+a_{2}\omega_{2}\alpha +2k_{2}^{2}\alpha }{k_{2}^{4}},C_{8}=1$ we get from eq. (28):

(29) $$u\left(x,t\right)=1+\frac{{\omega_{1}}^{2}+3\alpha a_{1}\omega_{1}+6\alpha k_{1}^{2}}{\sqrt{2\alpha }k_{1}\left(\omega_{1}+3\alpha a_{1}\right)}\mathrm{sech}\left(\frac{m\eta_{1}}{2}\right)e^{\frac{m\eta_{1}}{2}}+\frac{{\omega_{2}}^{2}+3\alpha a_{2}\omega_{2}+6\alpha k_{2}^{2}}{\sqrt{2\alpha }k_{2}\left(\omega_{2}+3\alpha a_{2}\right)}\mathrm{sech}\left(\frac{n\eta_{2}}{2}\right)e^{\frac{n\eta_{2}}{2}}\text{.}$$

If $C_{5}=\frac{\frac{1}{3}{\omega_{1}}^{2}+a_{1}\omega_{1}\alpha +2k_{1}^{2}\alpha }{k_{1}^{4}},C_{6}=-1,C_{7}=\frac{{\omega_{2}}^{2}+3a_{2}\omega_{2}\alpha +6k_{2}^{2}\alpha }{3k_{2}^{4}},C_{8}=1$ then from eq. (28):

(30) $$u\left(x,t\right)=1+\frac{{\omega_{1}}^{2}+3\alpha a_{1}\omega_{1}+6\alpha k_{1}^{2}}{\sqrt{2\alpha }k_{1}\left(\omega_{1}+3\alpha a_{1}\right)}\mathrm{sech}\left(\frac{m\eta_{1}}{2}\right)e^{\frac{m\eta_{1}}{2}}-\frac{{\omega_{2}}^{2}+3\alpha a_{2}\omega_{2}+6\alpha k_{2}^{2}}{\sqrt{2\alpha }k_{2}\left(\omega_{2}+3\alpha a_{2}\right)}\text{cosech}\left(\frac{n\eta_{2}}{2}\right)e^{-\frac{n\eta_{2}}{2}}\text{.}$$

If $C_{5}=\frac{\frac{1}{3}{\omega_{1}}^{2}+a_{1}\omega_{1}\alpha +2k_{1}^{2}\alpha }{k_{1}^{4}},C_{6}=-1,C_{7}=\frac{\frac{1}{3}{\omega_{2}}^{2}+a_{2}\omega_{2}\alpha +2k_{2}^{2}\alpha }{k_{2}^{4}},C_{8}=-1$ then from eq. (28):

(31) $$u\left(x,t\right)=1-\frac{{\omega_{1}}^{2}+3\alpha a_{1}\omega_{1}+6\alpha k_{1}^{2}}{\sqrt{2\alpha }k_{1}\left(\omega_{1}+3\alpha a_{1}\right)}\text{cosech}\left(\frac{m\eta_{1}}{2}\right)e^{-\;\frac{m\eta_{1}}{2}}-\frac{{\omega_{2}}^{2}+3\alpha a_{2}\omega_{2}+6\alpha k_{2}^{2}}{\sqrt{2\alpha }k_{2}\left(\omega_{2}+3\alpha a_{2}\right)}\text{cosech}\left(\frac{n\eta_{2}}{2}\right)e^{-\;\frac{n\eta_{2}}{2}}\text{.}$$

**Case-III:** For $a_{0}=-1$ then the remaining equations is gives us:

(32) $$f_{1}^{\prime }=\frac{3k_{1}^{2}}{3\alpha a_{1}-\omega_{1}}f_{1}^{\prime \prime }\text{.}$$

(33) $$f_{2}^{\prime }=\frac{3k_{2}^{2}}{3\alpha a_{2}-\omega_{2}}f_{2}^{\prime \prime }\text{.}$$

where $q=\frac{3\alpha a_{2}\omega_{2}-6\alpha k_{2}^{2}-{\omega_{2}}^{2}}{k_{2}^{2}\left(\omega_{2}-3\alpha a_{2}\right)}$ and $q=\frac{3\alpha a_{2}\omega_{2}-6\alpha k_{2}^{2}-{\omega_{2}}^{2}}{k_{2}^{2}\left(\omega_{2}-3\alpha a_{2}\right)}$.

If we insert eq. (32) and eq. (33) in eq. (6) and eq. (7) then we get the following value respectively.

(34) $$f_{1}=\frac{C_{9}3k_{1}^{4}}{{\omega_{1}}^{2}+6\alpha k_{1}^{2}-3\alpha a_{1}\omega_{1}}\;e^{p\eta_{1}}+C_{10}\text{.}$$

(35) $$f_{2}=\frac{C_{11}3k_{2}^{4}}{{\omega_{2}}^{2}+6\alpha k_{2}^{2}-3\alpha a_{2}\omega_{2}}\;e^{p\eta_{2}}+C_{12}\text{.}$$

Now from eq. (5), eq. (34) and eq. (35), with $a_{1}=\pm \sqrt{\frac{2}{\alpha }}k_{1}$, $a_{2}=\pm \sqrt{\frac{2}{\alpha }}k_{2}$ we get,

(36) $$u\left(x,t\right)=-1\pm \frac{\sqrt{\frac{2}{\alpha }}\frac{3k_{1}^{3}}{3\alpha a_{1}-\omega_{1}}C_{9}e^{p\eta_{1}}}{\frac{C_{9}3k_{1}^{4}}{{\omega_{1}}^{2}+6\alpha k_{1}^{2}-3\alpha a_{1}\omega_{1}}\;e^{p\eta_{1}}+C_{10}}\mp \frac{\sqrt{\frac{2}{\alpha }}\frac{3k_{2}^{3}}{3\alpha a_{1}-\omega_{1}}C_{11}e^{p\eta_{2}}}{\frac{C_{11}3k_{2}^{4}}{{\omega_{2}}^{2}+6\alpha k_{2}^{2}-3\alpha a_{2}\omega_{2}}\;e^{p\eta_{2}}+C_{12}}\text{.}$$

where $p=\frac{3a_{1}\omega_{1}\alpha -6k_{1}^{2}\alpha -{\omega_{1}}^{2}}{k_{1}^{2}\left(\omega_{1}-3a_{1}\alpha \right)},q=\frac{3a_{2}\omega_{2}\alpha -6k_{2}^{2}\alpha -{\omega_{2}}^{2}}{k_{2}^{2}\left(\omega_{2}-3a_{2}\alpha \right)}$ and $\omega_{1},k_{1},C_{9},C_{10},\omega_{2},k_{2},C_{11},C_{12}$ are arbitrary constants.

If $C_{9}=\frac{\frac{1}{3}{\omega_{1}}^{2}-\alpha a_{1}\omega_{1}+2\alpha k_{1}^{2}}{k_{1}^{4}},C_{10}=1,C_{11}=\frac{\frac{1}{3}{\omega_{2}}^{2}-\alpha a_{2}\omega_{2}+2\alpha k_{2}^{2}}{k_{2}^{4}},C_{12}=1$ we get from eq. (36),

(37) $$u\left(x,t\right)=-1+\frac{{\omega_{1}}^{2}-3\alpha a_{1}\omega_{1}+6\alpha k_{1}^{2}}{\sqrt{2\alpha }k_{1}\left(3\alpha a_{1}-\omega_{1}\right)}\mathrm{sech}\left(\frac{p\eta_{1}}{2}\right)e^{\frac{p\eta_{1}}{2}}+\frac{{\omega_{2}}^{2}-3\alpha a_{2}\omega_{2}+6\alpha k_{2}^{2}}{\sqrt{2\alpha }k_{2}\left(3\alpha a_{2}-\omega_{2}\right)}\mathrm{sech}\left(\frac{q\eta_{2}}{2}\right)e^{\frac{q\eta_{2}}{2}}\text{.}$$

If $C_{9}=\frac{\frac{1}{3}{\omega_{1}}^{2}-a_{1}\omega_{1}\alpha +2k_{1}^{2}\alpha }{k_{1}^{4}},C_{10}=-1,C_{11}=\frac{\frac{1}{3}{\omega_{2}}^{2}-a_{2}\omega_{2}\alpha +2k_{2}^{2}\alpha }{k_{2}^{4}},C_{12}=1$ then from eq. (36),

(38) $$u\left(x,t\right)=-1-\frac{{\omega_{1}}^{2}-3\alpha a_{1}\omega_{1}+6\alpha k_{1}^{2}}{\sqrt{2\alpha }k_{1}\left(3\alpha a_{1}-\omega_{1}\right)}\text{cosech}\left(\frac{p\eta_{1}}{2}\right)e^{-\;\frac{p\eta_{1}}{2}}+\frac{{\omega_{2}}^{2}-3\alpha a_{2}\omega_{2}+6\alpha k_{2}^{2}}{\sqrt{2\alpha }k_{2}\left(3\alpha a_{2}-\omega_{2}\right)}\mathrm{sech}\left(\frac{q\eta_{2}}{2}\right)e^{\frac{q\eta_{2}}{2}}\text{.}$$

If $C_{9}=\frac{\frac{1}{3}{\omega_{1}}^{2}-a_{1}\omega_{1}\alpha +2k_{1}^{2}\alpha }{k_{1}^{4}},C_{10}=-1,C_{11}=\frac{\frac{1}{3}{\omega_{2}}^{2}-a_{2}\omega_{2}\alpha +2k_{2}^{2}\alpha }{k_{2}^{4}},C_{12}=-1$ then,

(39) $$u\left(x,t\right)=-1-\frac{{\omega_{1}}^{2}-3\alpha a_{1}\omega_{1}+6\alpha k_{1}^{2}}{\sqrt{2\alpha }k_{1}\left(3\alpha a_{1}-\omega_{1}\right)}\text{cosech}\left(\frac{p\eta_{1}}{2}\right)e^{-\;\frac{p\eta_{1}}{2}}-\frac{{\omega_{2}}^{2}-3\alpha a_{2}\omega_{2}+6\alpha k_{2}^{2}}{\sqrt{2\alpha }k_{2}\left(3\alpha a_{2}-\omega_{2}\right)}\text{cosech}\left(\frac{q\eta_{2}}{2}\right)e^{-\;\frac{q\eta_{2}}{2}}\text{.}$$

## 4 Numerical discussion

**Fig. 1:**
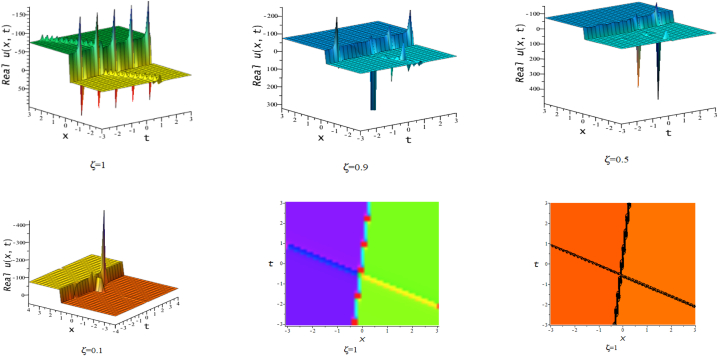
3-D, density and contour plots of eq. (20).

**Fig. 2:**
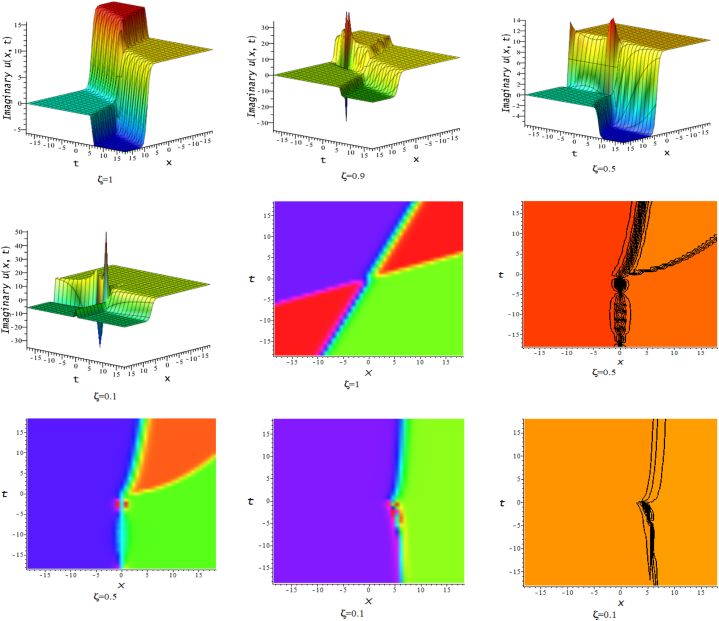
3-D, density and contour plots of eq. (21).

**Fig. 3:**
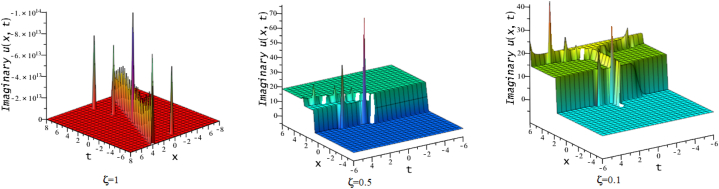
3-D plots of eq. (23) for ζ=0.1,ζ=0.5 and ζ=1:

**Fig. 4:**
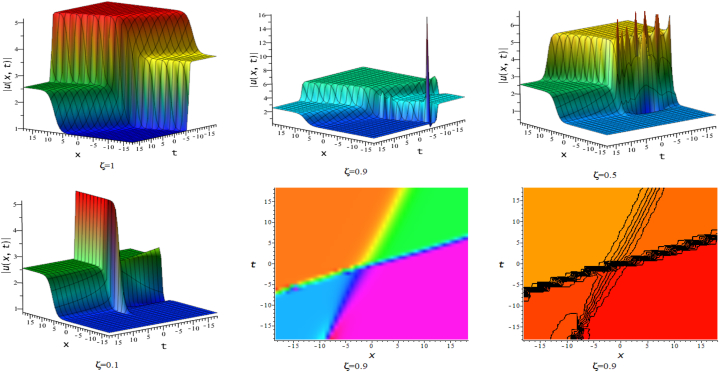
3-D, density and contour plots of eq. (28).

**Fig. 5:**
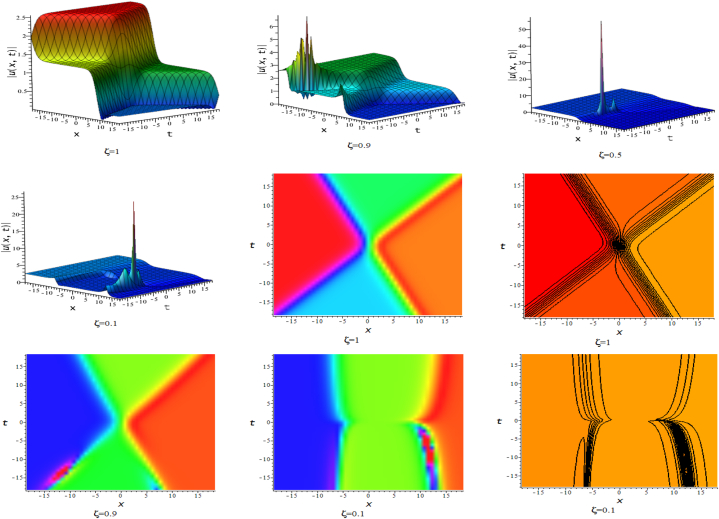
3-D, density and contour plots of eq. (29).

**Fig. 6:**
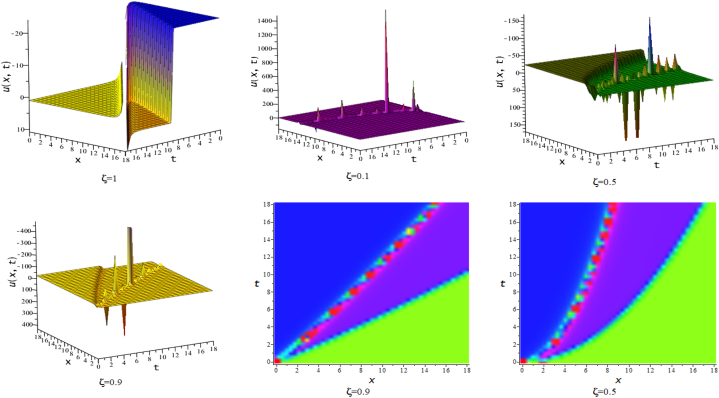
3-D, density and contour plots of eq. (30).

**Fig. 7:**
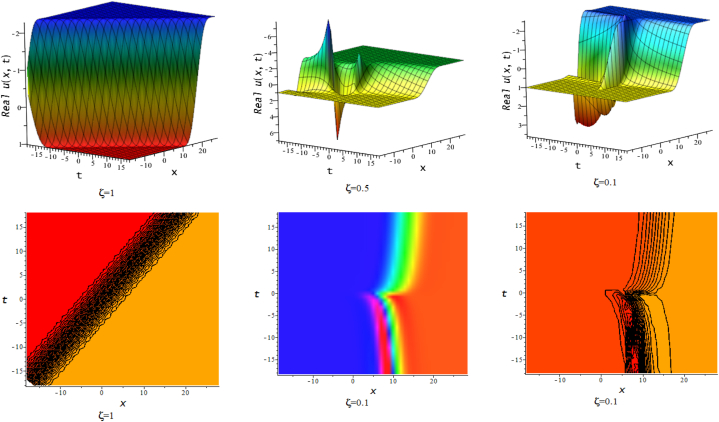
3-D, density and contour plots of eq. (31).

**Fig. 8:**
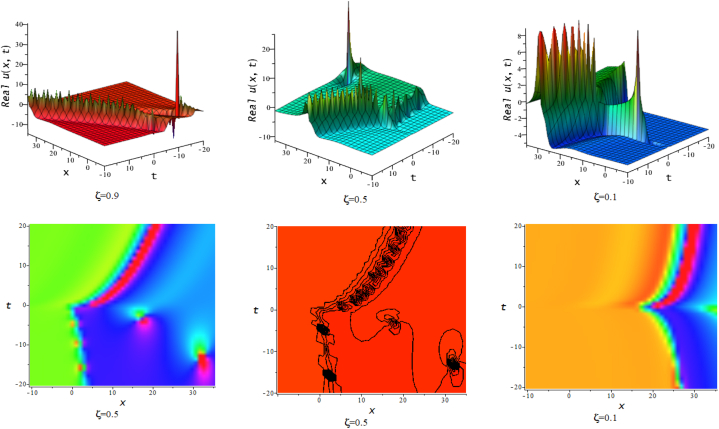
3-D, density and contour plots of eq. (36).

**Fig. 9:**
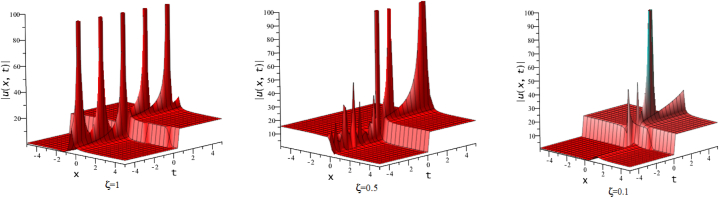
3-D plots of eq. (36) for ζ=0.1,ζ=0.5 and ζ=1. :

**Fig. 10:**
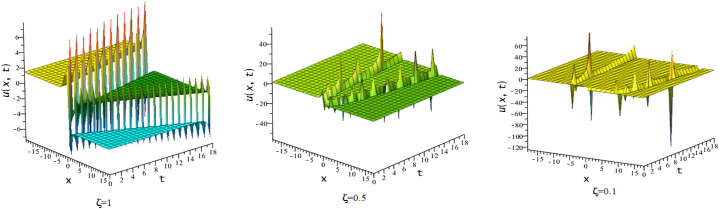
3-D plots of eq. (39) for ζ=0.1,ζ=0.5 and ζ=1. 5. Remarks and Comparison.

In this section, we discuss the behavior theoretically and graphically of the obtained solution with 3-D graph, corresponding density and contour plot for different values of ζ. Also deliberate the obtained solution under some conditions with numerical values. For the special value of free parameters under the conditions multi-kink soliton, multi-soliton solutions, interaction between kink and soliton, interaction between anti-kink and soliton, interaction of different soliton and double periodic soliton solutions are obtained. The interaction between a kink and a soliton solution derived via eq. (20) and its numerical presentation illustrated in Fig-1 for $k_{1}=-0.5,k_{2}=5,w_{1}=1,w_{2}=0.5,c_{4}=0.5,c_{3}=1,c_{2}=-3,c_{1}=-1,\alpha =3$ within the interval −18≤x≤18,−18≤t≤18. Here, we illustrated the effect of changing fractionality in the traveling wave at the interaction wave solutions, the observation concluded that the classical traveling wave variable expressed the multi-solitons in a kink wave structure, but as the classical wave tend to fractional form the soliton going to diminish into the kink structured only. Interaction of two-kink soliton wave arises via the solution in eq. (21) for $k_{1}=0.5,k_{2}=1,w_{1}=1.5,w_{2}=0.5,\alpha =-0.25$ in classical wave structured, which is completely elastic. But as traveling wave variable going to fractional form two-kink remain in the same structured and additionally few solitons visible in the physical structured, see Fig-2. Fig. 3 represented the interaction between kink and periodic solution with singularities for $k_{1}=7,k_{2}=1,w_{1}=1,w_{2}=0.5,\alpha =-4$. The solutions eq. (28) and eq. (29) have the similar behaviors like Fig. 2 represented the multi-kink. Overhead view of density plot and the 2-D contour plot of eq. (28) are drawn for the parameters $k_{1}=0.5,k_{2}=1,w_{1}=1.5,w_{2}=0.5,c_{5}=2,c_{6}=1,c_{7}=-5,c_{8}=-1,\alpha =1$ in Fig. 4 and the multi-kink soliton solution of eq. (29) for the value of the parameters $k_{1}=1,k_{2}=11,w_{1}=1,w_{2}=0.5,\alpha =1$ in Fig. 5. Fig. 6, represents the multi-soliton wave of evolution of the imaginary slice of eq. (30) for $k_{1}=0.5,k_{2}=0.5,w_{1}=1,w_{2}=0.5,\alpha =0.25$ with singularities. The lump-kink collision illustrated in Fig. 7 comes from eq. (31) with $k_{1}=k_{2}=w_{1}=w_{2}=1,\alpha =-0.25$ in classical case, due to change in the fractionality the structure deformed with multi-peaked into the lump-kink structure. Fig. 8 represented interaction of bell and periodic rogue waves from eq. (36) for $k_{1}=0.5,k_{2}=1,w_{1}=1.5,w_{2}=0.5,c_{9}=c_{10}=c_{11}=c_{12}=1,\alpha =-0.25$. In classical case the solutions exhibit such interaction of bell and periodic rogue waves, while fractionality reduced in comes to deform double bell wave. The interaction between the kink and soliton of eq. (36) for $k_{1}=10,k_{2}=0.5,\alpha =w_{1}=1,w_{2}=5$ are presented in the Fig-9**.** Fig. 10 represents the novel multi-soliton with singularities via eq. (39) for $k_{1}=1,k_{2}=-1,w_{1}=1,w_{2}=0.5,\alpha =1\text{.}$ :

In this division, we compare this work with existing published work [15,49,50]. The mention article applied different methods and find various soliton solutions but we applied NMSE method and investigate multi-soliton solutions and their interaction. In the former research, Kumar et al. [15] claimed the analytic solutions that include solutions based on rational, exponential, trigonometric and hyperbolic functions of CI model in the form of contained bright and dark solitons, singular and combined singular soliton profiles, periodic oscillating nonlinear waves, single and mixed singular soliton profiles, as well as kink-wave profiles. Akbar et al. [49] applied the first integral scheme and they derived the kink and singular kink waves, bright-dark and singular bell waves for CI model and discussed physical significant of obtained solutions with different parameters. Demiray et al. [50] derived anti-bell, kink and singular-kink wave of CI model by S-G expansion technique. Mahmood et al. [51] applied modified Khater scheme on CI model and obtained bright-dark, kink and periodic-oscillating waves. The accurate kink type solution of CI model was discovered by Habiba et al. [36] by improved Kudryashov technique. Mao [16] used the canonical-like transformation scheme and trial equation scheme to arrive at the exact solution to the CI problem in terms of the elliptic function. They used various methods with changing auxiliary equations only with single traveling wave variable. Besides this our main innovation is that, we introduced here the double waves solutions with different arbitrary wave speed in conformable fractional mode of CI model. The injected double wave traveling variables $\eta_{2}$ and $\eta_{1}$ with different wave speed gives collisions of two distinct wave structures. As a result, various interacted waves such as double kink (kink-kink collisions), lump-kink, periodic lump-kink, parabolic singular (in Fig. 10 for ξ=0.5), singular double kink interaction wave and double periodic waves (Fig. 6) are providing which is exceptional to the previous any works.

## 5 Conclusion, limitations and future works

### 5.1 Conclusion

In this study, we successfully implemented the new formation of MSE technique to solve fractional Chafee-Infante Equation. The proposed method is integrated multi-soliton solutions with their interaction of Chafee-Infante equation. On the parametric condition, notably the obtained solution is expressed as hyperbolic, exponential, trigonometric functions, and combine of them. To the best of our knowledge, the recent modification in the simple equation approach cannot be used to answer this equation. To help visualize how the equations behave dynamically, some figures are presented. From the above information the new form of modified simple equation approach seems to be simpler, faster, and easier for a computer to manage. This will encourage the extensive use of the equations in a sensible way. The obtained solutions may be substantial and imperative for scrutinizing the nonlinear phenomena rising in pragmatic physical sciences.

### 5.2 6.2 limitations

In this article, we investigate the multi-soliton solutions with their interaction of CI model. The NMSE technique are applied to obtained our required solutions. This method gives us a system of algebraic equation to giant their parameters which sometimes becomes impossible to solve by computational computer software.

### 5.3 6.3 Possible future works

We apply NMSE scheme on (1 + 1)-dimensional CI model with fractional differential form to derive interacted wave pattern. In future, we will apply the cofe transformation with Hirota bilinear technique and investigate N-soliton solutions, lump wave, breather wave and diverse type of interaction.

## Data availability statement

All the data associated this works are included in this manuscript.

## Funding statement

No founding is received for this work.

## CRediT authorship contribution statement

**Mohammad Mobarak Hossain:** Writing – review & editing, Writing – original draft, Software, Methodology, Formal analysis, Conceptualization. **Sushika Akter:** Validation, Methodology, Data curation, Conceptualization. **Md. Mamunur Roshid:** Writing – review & editing, Software, Methodology, Formal analysis. **Harun-Or-Roshid:** Writing – review & editing, Supervision, Methodology, Formal analysis, Data curation. **Md. Abu Naim Sheikh:** Supervision, Formal analysis, Data curation.

## Declaration of competing interest

The authors declare that they have no known competing financial interests or personal relationships that could have appeared to influence the work reported in this paper.
